# Development of an e-learning prototype for assessing occupational stress-related disorders: a qualitative study

**DOI:** 10.1186/s12909-019-1743-3

**Published:** 2019-08-09

**Authors:** Lieke Omvlee, Henk F. van der Molen, Ellen te Pas, Monique H. W. Frings-Dresen

**Affiliations:** 1 Amsterdam UMC, University of Amsterdam, Coronel Institute of Occupational Health, Netherlands Center for Occupational Diseases, Amsterdam Public Health research institute, PO Box 22660, 1100 DE Amsterdam, the Netherlands; 2 Amsterdam UMC, University of Amsterdam, Education Support, Amsterdam, the Netherlands

**Keywords:** Mental disorders, E-learning, Usefulness, Feasibility, Occupational physicians, Occupational stress-related disorders, Qualitative research

## Abstract

**Background:**

Occupational stress-related disorders are complex to diagnose and prevent, due to their multifactorial origin. We developed an e-learning programme aimed at supporting occupational physicians when diagnosing and preventing occupational stress-related disorders. In order to explore the extent to which a developed e-learning prototype was perceived as useful and feasible by occupational physicians, we executed a qualitative study.

**Methods:**

We conducted semi-structured, face-to-face interviews with fifteen occupational physicians, who were recruited using a combination of convenience and purposive sampling. Participants were shown a hard copy prototype of the e-learning programme, on which they were invited to comment in terms of perceived usefulness and feasibility. The interview data was transcribed verbatim and coded by two researchers using a content analysis approach.

**Results:**

Occupational physicians perceived e-learning as useful when it contributed to creating a full clinical picture and supported the diagnosis. Its structure had to support occupational physicians to work systematically. The programme had to be applicable to their daily practice and had to incorporate learning tools in order to increase the competences of occupational physicians. Feasibility was perceived to increase when the e-learning programme took less time to complete, when the quantity of written text was not too high, and when the user was guided and recertification points provided.

**Conclusions:**

An e-learning programme can be an asset in continuing medical education for occupational physicians when assessing occupational stress-related disorders. Perceived usefulness depended on the clinical picture, structure, practicality and the increasing of competences. Feasibility depended on text, time, structure and reward.

## Background

Common mental disorders, defined as non-psychotic mental-health problems, are prevalent in working populations: they affect one quarter of the population of Europe on an annual basis [1]. They cause decreased work ability and increased sickness absence [1, 2]. Examples of these disorders are depression, anxiety disorders and burnout. Several studies have shown that psychosocial working conditions affect mental health [1]. Moreover, developments such as increased work pace and the increased use of information and communication technology increase the demands on the mental functions of workers [3]. As such, psychological distress is widespread in the working population [3]. When these mental health problems become clinically relevant, they may be called stress-related disorders [3]. However, consensus has not been achieved among occupational physicians (OPs) when it comes to assessing the work-factors associated with occupational stress-related disorders [3]. In order to increase the knowledge and competences to diagnose and prevent occupational stress-related disorders (OSRDs), a guide for diagnosing and preventing OSRDs has been developed for Dutch OPs, which was updated in February 2016. It is based on a systematic review regarding work-related risk factors and currently consists of six steps [4] that OPs are encouraged to apply when diagnosing, reporting and preventing OSRDs (Table 1). OPs are encouraged to apply the six steps to obtain a structured evaluation of a worker, collect the necessary data and address preventive advice [4].

**Table 1:** Six step guideline to assess and prevent occupational diseases (ref [4])

Step 1. Determination of diagnosis OSRD	
Step 2. Determine relationship with work	
Step 3. Determine the nature and level of the causative exposure	
Step 4. Consider other possible explanations and the role of individual susceptibility	
Step 5. Conclusion and reporting	
Step 6. Preventative measures and interventions	

To support the implementation of the six steps, an e-learning programme has been developed. E-learning has become internationally recognized as a valuable learning method for health care professionals [5]. Tarpada et al. [6] define e-learning as the use of internet-based resources for teaching and learning purposes. Compared to traditional face-to-face education, e-learning has two main advantages. First of all, users have logistic freedom, since e-learning can take place at any place and time. Secondly, it has pedagogical advantages. Examples are the possibility to adapt to a user’s learning style by adjusting the content and following the user’s learning pace, as well as the possibility of repeating parts of the programme [7, 8]. An e-learning programme might therefore contribute to increasing and optimizing the OP’s knowledge of diagnosing and preventing OSRDs. The e-learning consisted of four parts, namely 1) A pre-test, consisting of multiple choice questions; 2) An explanation of the six steps in the updated guideline for stress-related disorders; 3) Three cases, in which the six steps in the guideline are applied in an interactive manner; 4) A post-test, consisting of multiple choice questions. Performance was tested by assessing the OPs’ skills when it came to diagnosing OSRDs in the pre- and post-test. However, our study focused on the parts two and three. The two main parts of the developed e-learning programme consist of an explanation of the six steps in the guide and three cases. In the first part, an explanation is provided about the way to diagnose a stress-related disorder, as well as its work-relatedness. This part also includes several evidence-based risk factors in the workplace that can have a causal effect on the occurrence of OSRDs, such as an imbalance in effort and reward and/or organizational injustice [9–12]. This is followed by an elaboration of the nature and level of causal exposure, the role of individual sensitivity, the decision about whether or not there is an occupational disease and the initiation and evaluation of preventative measures and interventions. In the three cases that follow, the six steps are put into practice. The user is guided, based on the six steps, through a case in which a patient visits an occupational physician and gives an explanation of his or her complaints, working conditions and personal life. Based on the information given by the patient, complemented by information about the six steps, users (i.e. OPs) diagnose each case and determine whether or not it is work-related. Finally, a suitable intervention is shown. Each choice the users make is presented as a test question, on which they receive feedback.

The e-learning was not tailored to a specific group of OPs (i.e. younger or older, experienced or starting). As such, the target group consisted of all OPs in The Netherlands. The overall purpose of the e-learning program was to increase OPs knowledge of and compliance with the six steps included in the evidence based guide.

Before implementation, it is important to evaluate a user’s perception of and attitude towards the e-learning programme so it can be optimized [5]. Cheng [13] states that perceived usefulness is an important determinant for the user’s intention to ultimately adopt a system. It refers to the user’s perspective on whether the e-learning programme is a useful learning tool [13]. Studying feasibility is a way to determine the acceptance or discarding of an intervention [14]. Establishing feasibility and perceived usefulness is frequently used to evaluate electronic programs within health care, and to decide whether or not to implement them [15–17]. In this study we aimed to determine whether the e-learning was perceived as useful and feasible. Perceived usefulness was defined in terms of whether OPs think the e-learning programme supports them in diagnosing and preventing OSRDs. Feasibility was taken into account as the factors that influence whether or not the e-learning programme would be followed by OPs in the future. We addressed the following research question: ‘To what extent is the e-learning programme about diagnosing and preventing occupational stress-related disorders perceived as useful and feasible by occupational physicians?’

## Methods

### Research design

A qualitative approach was taken, consisting of fifteen semi-structured face-to-face interviews about the perceived usefulness and feasibility of the e-learning programme. The interview questions, including a checklist of topics to which perceived usefulness and feasibility were applied, are listed in Table 2. This qualitative study was reported in line with the COREQ Checklist (COnsolidated criteria for REporting Qualitative research) [18].

**Table 2:** Interview questions and checklist

Perceived usefulness	
1. Does this e-learning programme support you in determining OSRDs and in initiating preventative actions? Why?	
2. Do you perceive this e-learning programme as useful? Why?	
Feasibility	
1. Is it practically feasible to complete this e-learning programme? Why?	
• Ask about perceived usefulness per step (perhaps elaborate “diagnosing”, “establishing the cause” and “prevention” more thoroughly).	
• Practical applicability	
• Sufficient/missing information	
• Comprehensibility/difficulty of steps	
• Structure	
• Perceived usefulness of cases	
• Extra sources of information needed?	
• Lay-out	
• Time	
• Recertification	
• Previous knowledge	
• Inviting	
• Language	

### Participants and selection

Recruiting took place during a conference for OPs about occupational stress-related disorders. A total of 284 OPs attended this conference. Before commencement of the study, no relationship with the participants was established. Participants were recruited using a combination of convenience and purposive sampling. Variation in age, sex, employment and experience with diagnosing OSRDs were a priori selection criteria. The attending occupational physicians were given the opportunity to fill in an application form requesting information concerning the above criteria. Since 38 attendees handed in their form, there was an opportunity for further selection. A sample was taken based on age, sex and employment of members of the main association for OPs in the Netherlands. With regard to experience with diagnosing OSRDs, we created as much variation as the sample allowed. Besides, we interviewed two representatives from the OP association, to review the prototype from their perspective. One of these participants was experienced in implementing e-learning programmes for OP’s. Selected participants were approached by email and telephone, received an informed consent form and were notified about the purpose of the study, and about the interviewing procedure. One selected participant dropped out due to holiday plans, and was replaced by an equally suitable participant.

### Procedure

Fifteen semi-structured interviews were conducted by the first author (female, 25 years old, Junior Researcher, MSc. in Social Sciences). She already had experience with conducting semi-structured interviews. Before the start of the data collection, the interview was pilot-tested with the second author, H.M. The interviews were planned to last one hour. All interviews were conducted from July to September 2016 and took place at a location convenient for the participant, usually the interviewee’s workplace or home. During all the interviews, only the interviewer and participant were present in the room. Three interviews were interrupted by text messages or phone calls, all lasting less than two minutes. Every interview was recorded, after the guarantee of anonymity and signing of the informed consent form. No field notes were made. At the start of each interview, the interviewer briefly repeated the purpose of the study and the interviewing procedure. The participant was invited to comment and ask questions during the interview. No characteristics about the interviewer were shared with the participant before the start of the interview. Beforehand, all participants had received a selection of e-learning sheets by email. During the interview, a printed version of these sheets was shown to the interviewee, while the interviewer explained the content and asked several questions concerning usefulness and feasibility. The participant was shown e-learning sheets explaining the six steps, one case and several test questions. The interview questions covered the extent to which the e-learning prototype was perceived as useful and feasible. We assumed data saturation was achieved when no new perspectives on perceived usefulness and feasibility of the e-learning programme were expressed.

### Data analysis

From every recorded interview, parts that were relevant for answering our research question were transcribed verbatim and coded using MAXQDA12, a software program for analysing qualitative data. A content analysis approach was used to analyse the data [19]. In this study, all selected quotes concerned the perceived usefulness and feasibility of the e-learning programme. Firstly, an inductive method of coding was applied, during which all quotes regarding perceived usefulness and feasibility were highlighted and separated. This process was executed by the first and second author, L.O. and H.M. Secondly, the first author refined and reduced the codes and categorized them hierarchically into themes and subthemes, while constantly comparing, rereading and discussing the (sub) themes with the co-authors. Participants were not involved in checking the findings.

## Results

Fifteen OPs, aged from 43 to 66, were included in the study. Of those fifteen participants, eleven were male and four female. Twelve were employees and three were self-employed. The amount of experience with diagnosing OSRDs varied. Table 3 shows a detailed overview of participant demographics. Tables 4 and 5 show the themes and subthemes about perceived usefulness and feasibility (respectively) that were identified from the interview data. The categories are illustrated by quotes.

**Table 3:** Characteristics of study sample of occupational physicians

Age	#Participants
40–50	3
50–60	7
60–70	4
Missing value	1
Sex	
Male	11
Female	4
Experience in diagnosing OSRD	
None	4
Some	6
A lot	5
Employment	
Employee at health service	12
Self-employed	3

**Table 4:** Themes and quotes about perceived usefulness

Theme	Sub-theme	Sub-sub-theme	Examples of relevant quotes
Diagnosing and preventing occupational diseases	Constructing full clinical picture		‘In that sense I have the urge to explore the whole picture, like, what is the picture, what do I know about the work and what’s not an issue, I think that system is easier to implement, (…).’ (int. 10) ‘(…) and I think the power of e-learning is, to, from the level of the book, from text and lists of questions, to come to a, to that picture. (...) If, yes, that’s what I missed in this (…).’ (int. 1)
	Structure contributes to working systematically		‘No yes, it goes through everything very systematically and (…) that does work in your consideration, so I think it works.’ (int. 7) ‘Its use is that you consider these steps more closely and, uh, this gives it a bit of structure. Uh, that you have a, uh, what do you call it, a decision tree that you can sort of run through.’ (int. 10)
	Practicality		‘To start with that makes me think, uh, (…) now then, it’s not very specific.’ (int. 7) ‘Yes. So it’s always good to have a list of sample questions (…) so you can carry on along those lines.’ (int. 8)
	Increasing competences	Reconsider own approach	‘Well, it helps me (…) in, yeah, the question, are you doing it good enough, like, like it helps to read it through again and then I think, well, I’m not that far off the mark.’ (int. 4) ‘And, well, I had another chance to think about it (…). An e-learning programme, that helps you do that (…).’ (int. 8)
		Gain and test knowledge	‘I think it’s better that you learn for yourself here, to make distinctions and to get it clear for yourself, uh, those are the symptoms, and then you can look at if it’s getting better or worse (…).’ (int. 6) ‘I think that too, too pampering. Yes, I think an occupational physician, (…) he should come up with his own questions, to get to the bottom of things.’ (int. 15) ‘No, but I think it’s good that it includes a case study, so you like, get a chance to practice behind the screen (…).’ (int. 12)

**Table 5:** Themes and quotes about feasibility

Theme	Sub-theme	Examples of relevant quotes
Time	Feasibility	‘That’s normal, and, it might take two hours, that’s fine, but not more than two hours, that’s too much.’ (int. 14) ‘But, let me put it like this, the shorter it is, the easier it is for people to do it alongside something else, right.’ (int. 9) ‘No I think a one-hour module, yes most e-learning is like that, that should be do-able (…).’ (int. 15)
Text	Quantity	‘When I read this, then I think there I go – then I quit! And I did quit, ‘cause I thought, I’m not going to read all this, just see how much text it is!’ (int. 14) ‘I don’t think it’s too much text, it’s not like you think, god where do I have to read now, no I really thought, like I said, I got stuck into it, yesterday and I thought, it’s really do-able.’ (int. 2)
	Comprehensibility	‘Cause for some sentences then I have to think three times, what does it mean, and it’s totally correct. It’s like, you can’t, no-one can, misinterpret it. Totally agree. But it’s so difficult to read.’ (int. 1) ‘Now this is where we give up of course (…) well, not me, WE give up, huh, ‘cause the odds ratio et cetera (...).’ (int. 13)
Structure	Guiding the user	‘(…) so you get taken by the hand, really clearly, step by step, through the guideline (…).’ (int. 9) ‘Cause sometimes I felt, while I was reading through it, it was difficult, like, where am I and what should I do.’ (int. 12)
Reward	Recertification point	‘Yes, don’t have to do that. Enough points, yes.’ (int. 11) ‘Yes, that’s an added incentive, but not the, not the, yes. No, not the deciding factor.’ (int. 7) ‘I think, I do additional training, additional e-training, it’s only good for one thing. Points.’ (int. 1)
	Inviting	‘Yeah, it certainly made me curious this morning (…).’ (int. 4) ‘And then I think like, I want to, know what the, I read it right through to the end, because I think, I want the result. (…) Yes I did it in one go too.’ (int. 6)

### Perceived usefulness

For perceived usefulness, all sub-themes were related to the theme ‘diagnosing and preventing occupational diseases’. Within this theme, four sub-themes were identified: (1) constructing full clinical picture, (2) structure contributes to working systematically, (3) practicality and (4) increasing competences (Table 4). Several OPs emphasized the importance of a full clinical picture constructed during consultations. This particular approach was described as emerging from their own intuition and professionalism, which generally resulted from their years of experience. The participants who relied on this approach generally perceived the e-learning programme as less useful, since its structure and the six steps on which it was based did not suit their intuitive way of diagnosing and preventing OSRDs, or the personal approach or system they had developed over the years. For instance, one participant described the power of e-learning as a way to support the construction of a full clinical picture, but did not think there was adequate support for his approach in this e-learning programme. He wanted to “*explore the whole picture*” and found his own approach “*easier to implement*”. The participants who emphasized the construction of a full clinical picture generally had an opposite view from the OPs who brought up the second sub-theme, “structure contributes to working systematically”. The participants who talked about this second topic described the structure offered in the e-learning programme as one of its main useful aspects, since they were in need of a structured method to diagnose and prevent OSRDs. For instance, one OP reported how the e-learning programme systematically goes through the topic, which helps in decision-making processes about OSRDs: “*Its use is that you consider these steps more closely and this gives it a bit of structure*.” The third sub-theme, the practicality of the e-learning programme, was also considered an important aspect of its usefulness. This topic was mainly about how the e-learning programme matched and contributed to the day-to-day practice of OPs. The content and features of the e-learning programme had to be specific, applicable and practical to be useful. For example, one participant found the chapter about good practices for prevention “*not very specific”*. The fourth sub-theme that was identified from the data was the possibility to increase one’s competences with regard to diagnosing and preventing OSRDs. Some OPs reported how the e-learning programme made them reconsider their own approach, like a participant who stated that the e-learning programme helped him establish whether his own approach was good enough and also said “*it helps to read it through again and then I think, well, I’m not that far off the mark.*” Within this sub-theme, a further-sub-theme emerged: “gaining and testing of knowledge”. Participants often brought up this topic in relation to the test questions and the cases. While some participants described the test questions as a useful way to test their knowledge or structure their thoughts, others considered them too easy, or even “*pampering”*. The cases were generally perceived as useful because they offered the user an opportunity to *“practice behind the screen”.*

### Feasibility

Four themes related to feasibility were identified: (1) time, (2) text, (3) structure and (4) reward (Table 5). When considering a feasible time for completing the e-learning programme, participants mentioned durations from a maximum of one hour to a maximum of two hours. A more general statement was that feasibility would increase if the e-learning programme took less time to complete, since *“the shorter it is, the easier it is for people to do it alongside something else”*. The second theme, “text”, consisted of two sub-themes: “quantity” and “comprehensibility”. The first was about the amount of text presented in the e-learning programme. Many OPs considered this too large, which had an adverse effect on feasibility. One participant even stated she had quit reading, saying *“I’m not going to read all this, just see how much text it is!”* Another remark was that some sentences were formulated in a way that was too abstract or scientific, resulting in the sub-theme “comprehensibility”. For instance, an OP stated he had to *“think three times*” about the meaning of the text. The theme “structure” was mainly about guiding the user through the e-learning programme, which was considered an important factor to increase feasibility. Opinions on this topic varied from positive statements (“*so you get taken by the hand, really clearly, step by step, through the guideline*”) to OPs who described some difficulty while reading. The fourth and final theme that was identified from the data was “reward”, consisting of the sub-themes “recertification point” and “inviting”. Firstly, some OPs stated that the recertification point as a reward was important, or even crucial, for following the e-learning programme. One of the OP’s even described it as the only reason, saying “*additional e-training, it’s only good for one thing. Points.”* However, others said they already earned enough points or were more intrinsically motivated and did not consider this reward to be a prevailing factor for feasibility (*“that’s an added incentive, but not the ( …*) *deciding factor”*). When it came to the sub-theme “inviting”, according to some OPs an inviting e-learning programme contributes to feasibility if it is fun to do, or if it arouses curiosity in the user, like a participant who said “*it certainly made me curious this morning*.”

## Discussion

Several studies about e-learning confirm the themes identified in our research [20–22]. Robson [21] mentions practicality, the reconsideration of one’s own approach, structure and the gaining and testing of knowledge as important topics. Asarbakhsh and Sandars [22] focus on text, as well as the required time and effort it takes to follow an e-learning programme. As such, our findings can be used as points of interest for the development of future e-learning programmes concerning the assessment of work-related diseases by OPs. In this project, the e-learning programme has been modified on several points based on the participants’ comments: the amount of written text has been reduced, some parts have been rewritten to enhance comprehensibility, and extra attention has been paid to adapt the e-learning programme to the day-to-day practice of OPs. However, the theme “constructing full clinical picture” was difficult to take into consideration, because OPs reporting on the ‘full clinical picture’ often criticised compliance with guidelines since they feel it is a ‘cookbook’ without possibilities to deviate from it. Therefore, it was complicated to adjust the e-learning programme to cater for OPs who rely on their own expertise and use a different approach from the one in the e-learning programme. Contrary, we aimed to develop an e-learning to increase the knowledge and skills of OPs to assess and prevent OSRDs to comply with evidence-based guidelines. However, it does raise the question as to what might be a fitting learning method for these medical professionals. In order to also meet the needs of OPs who rely on their own, more intuitive system, a next step might be to adjust the e-learning according to their existing knowledge. For OPs with doubts concerning the use of guidelines, other (training) interventions could be useful to improve compliance with existing guidelines. E.g. feedback from peers, a form of collaborative education, or case reports may enhance knowledge and skills for work attribution [23, 24]. This aspect is also emphasized by Sinclair et al. [25], who state that the existing knowledge of the user should be taken into consideration. In order to meet the needs of every user, the e-learning programme can be personalized with test questions and cases that establish the current level of knowledge, followed by a module that is adapted to the individual user [25]. A multifaced knowledge translation as described by De Silva and colleagues, consisting of several educational activities could increase the personalization and therefore the learning efficiency [26]. However, such a high level of personalization was beyond the scope of this project. As for e-learning programmes in a later stage of development, research shows they contribute to knowledge and skills when it comes to implementing guidelines in healthcare [27, 28].

Although several studies deal with e-learning for occupational physicians [29–31], and the feasibility and/or usefulness of other electronic systems in health care [15, 17], most of these studies use a quantitative approach, measuring, for instance, acceptability for the user of an electronic program by sending out surveys [15]. Although quantitative measuring can result in clear user feedback on predetermined categories, our qualitative approach led to a focus on the user’s perspective and themes that were identified using an inductive approach. This was particularly useful since it resulted in suggestions for the improvement of the e-learning programme that we had not thought of beforehand, such as increasing the comprehensibility of the text. It gave us more detailed insight into the reasons for the diverse opinions, and thus into the needs of the participants with respect to the e-learning programme. However, a limitation of this study is that triangulation in the method of data collection was not used. Combined research methods can increase the ability to interpret the findings.

A strength of this study was the composition of the sample, which was a reflection of the members of the Dutch association for OPs. Furthermore, our sample was varied in terms of experience with diagnosing OSRDs, resulting in an overall diversity in the identified themes. Taking different themes into account enhanced the applicability of the e-learning programme to the population of its potential users. All defined topics were discussed and data saturation, which occurred after about eight interviews, was achieved over the different aspects of the e-learning programme. We applied a purposive sampling approach, but our approach may have excluded OPs who are not interested in e-learning [32]. This may have resulted in a bias towards OPs who were generally positive about e-learning.

Overall, e-learning can be an effective learning tool for health care professionals as part of continuing education, since it increases their knowledge [20, 21, 29] and is a promising tool with regard to improvements in practice [20, 21]. For example, Hugenholtz et al. [29] have shown that e-learning increases the knowledge of OPs concerning the topic of mental health. However, it is still unclear to what extent it supports the OPs’ professional practice when it comes specifically to the diagnosing and preventing of OSRDs. Future research could provide more insight into the effectiveness of this e-learning with regard to practice.

## Conclusions

This study gives an insight into the perceived usefulness and feasibility of an e-learning programme concerning occupational stress-related disorders for occupational physicians. Within perceived usefulness, there was a distinction between OPs who were looking for more structure in this process and OPs who relied on their own professionalism and expertise. Furthermore, the practicality of the e-learning programme and its feature to gain and test the user’s knowledge were two main themes. Feasibility depended on time, text, structure and reward. The amount of written text appeared to be a major obstacle to completing the e-learning programme. For some OPs, recertification was an incentive to following the e-learning programme, whereas others were more intrinsically motivated.

## Data Availability

The qualitative data used and analysed during the current study are available from the corresponding author at reasonable request.

## Abbreviations

OP: Occupatioal physician
OSRD: Occupational stress-related disorder
